# Virus-like particles derived from bacteriophage MS2 as antigen scaffolds and RNA protective shells

**DOI:** 10.2217/nnm-2023-0362

**Published:** 2024-04-17

**Authors:** Antonina Naskalska, Jonathan Gardiner Heddle

**Affiliations:** 1 Malopolska Centre of Biotechnology, Jagiellonian University, Krakow, 30-392, Poland; 2 Department of Biosciences, Durham University, Durham, DH1 3LE, UK

**Keywords:** bionano, capsid, drug delivery, phage, protein cage, protein engineering, RNA delivery, RT-PCR, vaccines

## Abstract

The versatile potential of bacteriophage MS2-derived virus-like particles (VLPs) in medical biotechnology has been extensively studied during the last 30 years. Since the first reports showing that MS2 VLPs can be produced at high yield and relatively easily engineered, numerous applications have been proposed. Particular effort has been spent in developing MS2 VLPs as protective capsules and delivery platforms for diverse molecules, such as chemical compounds, proteins and nucleic acids. Among these, two are particularly noteworthy: as scaffolds displaying heterologous epitopes for vaccine development and as capsids for encapsulation of foreign RNA. In this review, we summarize the progress in developing MS2 VLPs for these two areas.

## Plain language summary

Hollow, nanosized protein particles have many potential uses. If they can be appropriately engineered, they may for example be able to carry therapeutic cargoes to diseased cells or be used as a vaccine where appropriate antigens are mounted on their external surface. Many viruses offer a ready-made protein particle, the capsid, which can be made hollow by exclusion of the viral genetic material. MS2 is a virus that targets bacteria – a bacteriophage – which is well characterized and has been developed over many years for a number of applications. It has particular promise for development as a vaccine and for RNA delivery, both of which are reviewed here.

## Abstract (plain language summary)

A number of virus-like particles (VLPs) are known to be effective immunogens and in some cases have been developed into clinically approved vaccines.

MS2 is an icosahedral ssRNA bacteriophage around 26 nm in diameter.

Immunogenic epitopes can be genetically engineered into the externally displayed AB loop of the capsid proteins.

MS2 VLPs displaying such epitopes have been shown to be capable of eliciting an immune response in mice.

Genetic fusion of identical coat protein monomers to form fused dimers stabilizes the resulting VLP but only one A/B loop of the resulting dimer can tolerate an insertion if stability is to be maintained.

Proof-of-principle studies have been carried out for MS2 modified to act as vaccines against HPV, foot-and-mouth virus and *Chlamydia trichomatis*.

The ability of MS2 VLPs to encapsidate their own encoding nucleic acid means that CPs with fused peptide can be used to ‘biopan’ a peptide library for binding to targets (e.g., antibodies) wherein the sequence encoding binding peptides can be easily recovered.

MS2 encapsidates its own RNA by binding to the ‘*pac* site’, a stem-loop structure.

MS2 VLPs can encapsidate foreign RNA sequences by fusion to a *pac* site sequence.

Fusion of target RNA to a copy of the *pac* site sequence with expression from the same transcript as the MS2 *CP* and the *maturase* avoids nonspecific RNA packing. The RNA is protected and stabilized and is known as armored RNA.

Armored RNA can be used as internal control standards in real-time-PCR.

Armored RNAs able to protect longer RNA sequences were developed through modification of expression vector strategy and *pac* site.

Both *in vivo* and *in vitro* RNA packaging approaches have been developed.

Foreign sRNAs and mRNA can be encapsidated within MS2 VLPs.

MS2 VLPs decorated with cell-penetrating peptides can deliver miRNAs to cells.

mRNA, being longer, is more challenging to package but this has been demonstrated.

## Graphical abstract

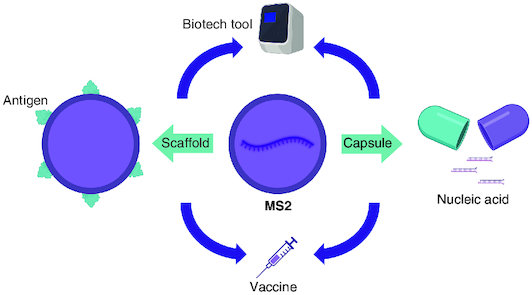

Bacteriophage MS2 has features and properties that make it attractive for a range of biotechnological and biomedical applications. Figure created with Biorender.com.

Viruses effectively deliver nucleic acid materials to cells. For this reason, replacing native genetic material with therapeutic nucleic acids or other cargoes has long been attractive and considerable research has been carried out in the area [1–3]. Bacteriophages (viruses that target bacteria) are of particular interest, being nonpathogenic for humans. Attractive features of such virus-based systems include the fact that heterologous DNAs and RNAs can be relatively easily incorporated into the bacteriophage genome and that peptide display on virus-like particles (VLPs) derived from bacteriophages is highly feasible meaning that these vehicles can be ligand-targeted to a specific cell type. In addition, available bacteriophages cover a wide range of sizes and shapes which may give different biodistribution properties, and they are typically easy to handle with good stability and easy to produce at large scales.

MS2 is an icosahedral ssRNA bacteriophage belonging to the *Leviviridae* family. Its capsid, measuring ∼26 nm in diameter, consists of 178 copies of coat protein (CP) and one copy of the maturation protein (A protein or maturase). These are encoded in the ∼3.6 kb RNA genome which also encodes a replicase, and lysis protein. During virus assembly the CP dimerizes and binds to a specific viral RNA hairpin (called the *pac* site). However, when the CP is expressed from a plasmid in a bacterial or yeast host, in absence of other viral elements, it self-assembles into empty capsids called VLPs. Analogous to the native MS2 virion, formation of the MS2 VLP involves dimerization of the CP and formation of an unstructured loop connecting the F and G β-strands of each subunit (known as the FG loop) [4]. Depending on the conformation of the FG loop, the CP monomer can exist in three conformations: A, B and C. Likewise, the resulting dimers adopt two forms: asymmetric (called A/B) or symmetric (called C/C) as depicted in Figure 1. Of note, MS2 VLPs are composed of 90 CP dimers (60 A/B dimers and 30 C/C dimers), thus compensating the lack of maturase protein present in the native virion.

**Figure 1.**
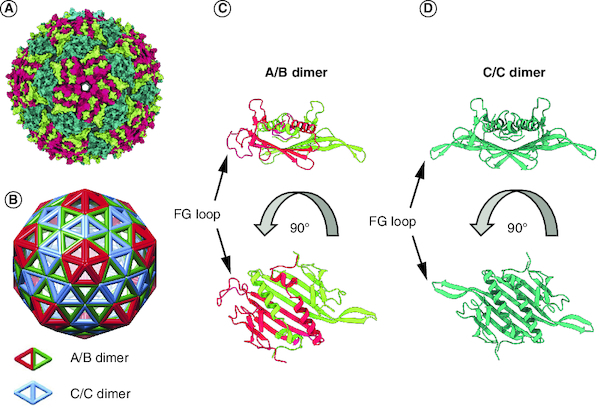
Structure of MS2 bacteriophage. **(A)** Crystal structure of MS2 (PDB 2MS2) [5]. CPs A, B and C are colored lime green, dark pink and coral blue, respectively. **(B)** Schematic representation of the structure of MS2 showing that CP forms dimers adopting two conformations: called A/B and C/C as indicated, adapted from Biela *et al.* published under a CC BY 4.0 license (http://creativecommons.org/licenses/by/4.0/) [6]. **(C & D)** Close-up orthogonal views of the structures of two dimer types represented as ribbons and colored as in panel **(A).** CP: Coat protein; PDB: Protein Data Bank.

Since the initial description of MS2 VLPs [7] they have been extensively characterized and much is now understood of the mechanisms governing the assembly process, the flexibility of the particle in terms of supporting structural modifications and possibility to exploit its internal cavity. Purification protocols have also been developed and refined. Many of these studies were pioneered over several decades by the groups of Peabody [4,8–12] and Stockley [13–19]. They have enabled further development of MS2 VLPs for the applications described below. Here we identify and review the development of MS2 VLPs as scaffolds displaying antigenic peptides for vaccine development and as capsids for encapsulation of foreign RNA.

## MS2 VLP as scaffolds displaying antigenic peptides

VLPs are known to be effective immunogens, a property that is exploited in a number of successful VLP-based vaccines. The most established examples include the human papillomavirus (HPV) vaccine and the hepatitis B vaccine [20]. The immunogenic efficacy of VLPs appears to be related to their morphological properties such as size, shape and the high-density display of multiple copies of antigens. This results in efficient uptake by antigen-presenting cells and subsequent stimulation of B lymphocytes for antibody production. Additionally, unlike other exogenous antigens, VLPs are cross-presented in association with both MHC class I and class II despite the absence of infection or intracellular replication. Consequently, they are highly effective at stimulating CD4^+^ T-helper and CD8^+^ cytotoxic T-lymphocyte responses. Finally, thanks to pathogen-associated molecular patterns originating from producer cells, VLPs can activate the innate immune system too. Because of this, VLPs may be considered as self-boosting antigen carriers and therefore may serve as ideal scaffolds displaying poorly immunogenic antigens [21]. Such chimeric VLPs are also an attractive option for multivalent vaccines, which display several different epitopes on one particle, raising the potential for ‘universal’ vaccines against multiple viral strains [22,23].

First attempts to incorporate immunogenic epitopes into MS2 VLPs were reported by Mastico and colleagues [15] who reasoned that the most distal loop present at the particle surface could accommodate foreign peptides. This loop corresponds to the N-terminal β-hairpin of the CP and is also referred to as the AB loop [24]. Using a genetic fusion approach, Mastico *et al.* were able to insert several viral and nonviral peptides of 8–24 amino acids length. Some variants of the produced chimeric MS2 VLPs were soluble and could be further purified and used to immunize mice. Effective production of high titer antibodies specific to the peptides displayed on the MS2 VLP was obtained, thereby providing a proof-of-concept for further development of such a system. This strategy was subsequently used to produce MS2 VLPs displaying malaria epitopes [25]. Specifically, an epitope from the immunodominant liver-stage antigen-1 of the malaria parasite *Plasmodium falciparum* was incorporated into MS2 VLPs. The ability to induce humoral and cellular-specific immune responses by this vaccine candidate was demonstrated in mice models. However, based on subsequent findings related to *Plasmodium* evasion of human immune response, other malaria antigens were targeted [26]. Eventually, after many years of efforts, an effective malaria vaccine, based on a VLP (derived from hepatitis B virus) displaying another antigen (RTS,S) has been developed and licensed (Mosquirix™, GSK, London, UK).

Structural studies of MS2 VLP have shown that genetic fusion of the two identical CP monomers results in the expression of properly folded CP single-chain dimers [10]. Moreover, it was found that this modification protects the protein against the destabilizing effects of amino acid substitutions and chemical denaturants, and thus stabilizes the resulting VLPs [12]. It is worth noting, however, that only one insertion of a foreign peptide into the single-chain dimer is tolerated if proper VLP assembly is to be achieved. Thus, the display density on the VLP is consequently reduced by half (from 180 copies to 90). Using this approach Peabody and colleagues managed to incorporate 6, 8 and 10 amino acid peptides originating from an HIV antigen into MS2 VLPs [27].

While MS2 has been investigated, the general field of VLP-based vaccine development has experienced great success with the approval of VLP vaccines protecting from infection of HPVs (Gardasil^®^, Merck & Co. [NJ, USA]), Cervarix^®^ (GSK) [28]. These VLPs are made of HPV major capsid protein L1 and are protective against several of the most common serotypes of the virus. However, the L1 protein is not conserved among all existing serotypes and thus L1-based vaccines do not confer enough cross-protection. On the other hand, the minor HPV capsid protein – L2 – is highly conserved among HPV types and therefore of interest in developing a broadly protective HPV vaccine. Unfortunately, the L2 protein, unlike L1, cannot form VLPs and as such must be displayed on carriers to ensure sufficient immunogenicity. Production of an MS2 VLP displaying HPV L2 antigens was first reported in 2012 by Tumban *et al.* [29] and further improved by the same group [30–32]. Remarkably, Tumban and coauthors chose a different location for epitope insertion: the gene coding for L2 peptide was fused to the N-terminus of the CP single-chain dimer. Interestingly, they observed that this location offered better cross-protection against diverse HPV serotypes [33]. They also tested insertions ranging from 12 to 27 amino acids, and created bivalent MS2 VLPs displaying 17–36 and/or 69–86 HPV L2 peptides, further increasing cross-reactivity [30,34]. Finally, they developed a formulation to enhance vaccine stability and clinical applicability and refined its optimal dose [35]. The efficacy of all produced MS2 HPV vaccines was validated in challenge studies in mice.

In 2015, the production of MS2 VLP-carrying epitopes for foot-and-mouth disease virus (FMDV) was reported [36]. Here, a 19 amino acid peptide (epitope peptide 141–160) was inserted into the AB loop of the single-chain dimer of CP. Proper assembly of the chimeric VLPs was demonstrated, but also challenge tests on guinea pigs and swine were carried out, showing that the vaccine could partially protect animals against FMDV. This vaccine candidate was improved 3 years later by elongating the immunodominant epitope with a motif shown to be essential in inducing FMDV neutralizing antibodies (epitope peptide 131–160) [37]. The authors of these studies not only demonstrated that their preparation could efficiently enhance antibody levels and cellular immune response compared with a commercialized synthetic peptide vaccine but also provided evidence that MS2 VLP can be used in larger mammals.

When Zika virus (ZIKV) emerged, a chimeric MS2 VLP displaying viral peptides was constructed. Six different ZIKV envelope protein fragments (up to 21 amino acids), theoretically predicted to be B-cell linear epitopes, were inserted into the N-terminus of the single-chain dimer of MS2 CP [38]. Mice immunized with a mixture of VLPs displaying ZIKV epitopes elicited anti-ZIKV antibodies. Although immunized mice were not protected against a high-challenge dose of ZIKV, their sera neutralized a low dose of ZIKV *in vitro*. The authors hypothesized that this could be because the epitopes displayed in MS2 VLP were linear and not conformational (as in the native virus). Similar observations were reported by Mogus and coauthors who displayed the linear epitope from HIV fusion peptide 8 on MS2 VLP via N-terminal fusion to the CP single-chain dimer. Mice immunized with this vaccine candidate exhibited high titers of fusion peptide 8-specific antibodies, albeit not correlating with neutralization or protection [39]. These findings perhaps indicate that for complex antigens requiring additional modifications (i.e., quaternary structure) genetic fusion to CP and subsequent expression in bacteria is not the optimal strategy. This can, however, be overcome by post-assembly attachment of such complex antigens. One example is the work showing successful tethering of biotinylated SARS-CoV-2 spike protein antigen to MS2 VLPs coated with streptavidin [40].

The most recent MS2 VLP-based vaccine candidate is the one displaying a *Chlamydia trichomatis* conserved epitope. The 9–10 amino acid peptides were inserted into the AB loop and immunization resulted in protection against a vaginal *Chlamydia* challenge in mice [41].

All published work cited in the section above (summarized in Table 1) readily shows that MS2 VLPs can act as universal antigen scaffolds with promising immunological potential. Also, taking into consideration the number of studies *in vivo*, they seem to exhibit a good safety profile – a feature considered to be essential in the field of vaccine development.

**Table 1.** MS2 as scaffolds for foreign peptide display.

Target	Insertion position	Peptide length (amino acid)	Immunogenicity testing	Ref.
Influenza virus, *Plasmodium falciparum*, human papillomavirus, HIV	AB loop	8–24	Mice	[15]
*Plasmodium falciparum*	AB loop	24	Mice	[25]
HIV	AB loop	6, 8, 10	Mice	[27]
Human papillomavirus	N-terminal	12–27	Mice	[29]
Foot-and-mouth disease	AB loop	19, 29	Guinea pigs, swine	[36]
Zika virus	N-terminal	21	Mice	[38]
HIV	N-terminal	8	Mice	[39]
*Chlamydia trichomatis*	AB loop	9–10	Mice	[41]

In the research area combining vaccine development and MS2 VLPs, there is one more interesting application: biopanning – a method to evaluate antigens in terms of their reactivity with antibodies. In general, this assay takes advantage of the fact the MS2 VLPs encapsidate the nucleic acid that encodes the CP and its guest peptide (as explained in the next section). This means that MS2 VLPs displaying a peptide library can be affinity-selected by interaction with the specific antibody (i.e., from the patient's serum) and its genetic sequences can be recovered and amplified by reverse transcription and PCR. As a consequence, this method allows researchers to pan an antigen in order to identify targets of antibody response or the opposite: to map the antibody repertoire against a defined antigen. Using this approach the specific neutralization-sensitive epitope RH5 [42] and the conformational epitope within blood-stage protein AMA1, both from *P. falciparum*, were identified [43]. Similarly, dengue virus epitopes recognized by antibodies elicited during acute infection were identified [44]. Importantly, the MS2 VLP can integrate the epitope identification and immunization functions into a single platform as products of affinity selection can be directly used as vaccines.

### MS2 VLP as capsids for encapsulation of foreign RNA

As already mentioned, in MS2 bacteriophage the packaging of RNA molecules is driven by the dimerization of CPs followed by a specific interaction with a stem-loop structure called the *pac* site. This stem loop is located at the 5′ of the replicase gene and its binding to CP triggers encapsidation during the assembly process of the MS2 virion, but also acts as a repressor for bacteriophage replication [7]. Interestingly, when the *pac* site sequence (19 nucleotides [nt]) is fused to a foreign sequence of interest, interaction with the MS2 CP expressed within the same cell is retained and thus packaging of the respective RNA molecule into MS2 VLP can be achieved [8]. Of note, the maturase also plays a role in the process: in the MS2 bacteriophage it interacts specifically with viral RNA [45] and it is assumed that it may facilitate the packaging of foreign RNA into MS2 VLPs. Since the first report describing this packaging strategy in MS2 VLPs, its efficiency has been improved by several modifications of the expression system, as discussed below.

### Armored RNA

**Figure 2.**
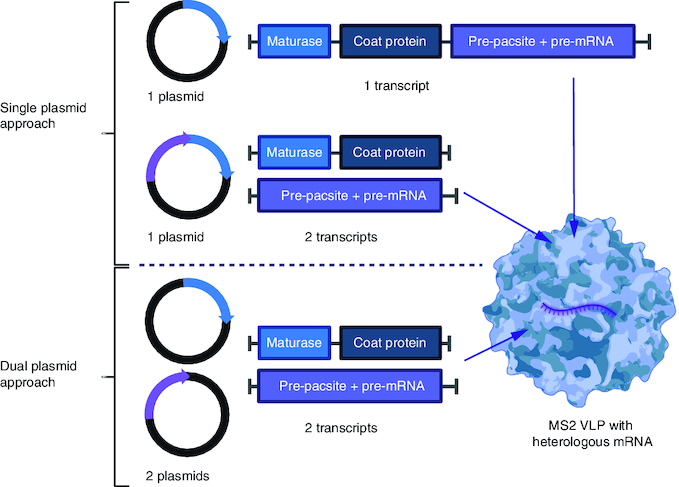
Comparison of expression systems used to encapsulate foreign RNA in MS2 virus-like particles. In the single-plasmid system (top) the maturase, coat protein and RNA sequence to be packaged are encoded on one transcript. Alternatively, maturase and coat protein are encoded on one transcript and the RNA sequence on another, both in the same plasmid. In the two-plasmid system (bottom) the *maturase*, coat protein and RNA sequence to be packaged are encoded on two separate transcripts, from two different plasmids. Figure created with Biorender.com. VLP: Virus-like particle.

After first demonstration that foreign RNA can be encapsidated inside MS2 VLP [8], the technology was named armored RNA (AR) and has been further developed and patented by du Bois and Pasloske [46]. These authors constructed a one-plasmid-driven packaging system. In this single-plasmid approach, the target RNA is fused to one copy of the *pac* site sequence and expressed from the same transcript as the MS2 *CP* and the *maturase* (Figure 2). It was claimed that such a design results in the optimal ratio of recombinant RNA to MS2 CP and thus avoids the nonspecific packaging of bacterial RNA, which was the major limitation of the initial system described by Pickett and Peabody. DuBois and Pasloske assumed that when *CP* is being translated from the same RNA that is to be packaged, its expression is regulated because once the concentration of CP is sufficiently high, it encapsidates the RNA from which it is being translated and thus prevents any further *CP* from being translated from that RNA [47]. Using this approach Pasloske produced AR containing the 172-base consensus sequence from the HIV type 1 *gag* gene and a consensus 412-base sequence from the 5′-non-coding region/core region of hepatitis C virus subtype 2b [46].

The AR is not only protected from ubiquitous nucleases, but also the concentration of the packaged RNA is controllable, which means that such particles can be used as standards (internal controls [ICs]) for detection or quantification of the corresponding native viruses. Two major advantages of using IC AR are that, contrary to naked RNA, they can be stored for several months at -20°C without losing integrity; and that the AR is processed identically as the tested sample during all steps of the quantification assay: it has to be extracted from the viral particle, reverse-transcribed and then PCR-amplified. As a consequence, using AR as IC allows for more accurate quantification of viral loads in tested samples and thus improved reliability of diagnostic assays. After being patented, the IC AR prepared in MS2 VLPs technology was further commercialized by Ambion Inc. (later Asuragen, TX, USA). In the next years, several different groups described the use of similarly produced enterovirus, HCV, hantavirus and a Crimean–Congo hemorrhagic fever virus AR in real time-PCR diagnosis of clinical specimens [48–51]. Remarkably, a smart improvement of this technology has been introduced by Cheng and colleagues who incorporated a histidine-tag (His-tag) into single-chain dimer CP thus simplifying the purification of particles using His-tag affinity [52].

It is worth noting that in all examples cited above, the length of RNA packaged in MS2 VLPs is less than 500 bases. Although most RT-PCR assays do not target RNA sequences longer than 500 bases, there are some advantages if longer target RNA sequences are packaged. For instance, in PCRs in which several primer pairs are used for the detection of different regions in the target gene. In addition, if long RNA sequences could be packaged, a single chimeric AR could serve as a standard for a variety of different assays designed for a one-step detection of multiple viruses. To meet this need Wei and coauthors proposed two modifications in the AR technology design that significantly improved the MS2 particle loading capacity [53]. First, they returned to the original idea of using a two-plasmid coexpression system in which the *CP* and the *maturase* were expressed from one plasmid while the target RNA containing the *pac* site was produced by a second plasmid (Figure 2). In order to ensure the optimal ratio of CP and the RNA to be produced, these two plasmids should have equivalent copy numbers. Additionally, plasmids should encode resistance to two different antibiotics, to be stably maintained together in the same bacterial host [53]. Second, they used a variant of the *pac* site in which uridine at position 5 was replaced by cytosine (called C5 variant), as it has been previously shown that this C5 variant exhibits 6–50-fold increased affinity to the CP [54]. Thanks to these two modifications Wei and colleagues managed to produce the AR comprising a 2248 nt foreign RNA sequence, which includes three SARS-CoV-1 fragments, one HCV fragment and two H5N1 fragments [53]. They also validated the obtained AR as IC for qualitative or quantitative detection by RT-PCR of the respective viruses. Next, the same group demonstrated that adding a single repetition of the *pac* site sequence (both wild type and C5 variant) in the one-plasmid expression system resulted in increased RNA packaging (from 1.2 kb to 1.8 kb) [55]. Finally, these authors improved the packaging efficiency even further by using a one-plasmid double expression plasmid, where *CP* and *maturase* are expressed from the first cloning site of pACYC Duet vector and target RNA with three copies of *pac* site sequence – from the second cloning site (Figure 2) [56]. In this and subsequently published work, long RNA fragments (up to 3 kb) were packaged in MS2 VLPs (Table 2) [56–59]. Interestingly, thanks to the mentioned modifications, Wei and coauthors exceeded the theoretical packaging limit of approximately 2.0 kb (taking into consideration that MS2 bacteriophage RNA genome is 3.6 kb and the sequence encoding the CP, the maturase and the *pac* site is 1.7 kb). They hypothesized that this particular setting (one-plasmid double expression and presence of doubled or tripled *pac* site) might benefit from an optimal ratio of CP/RNA and a cooperative binding of RNA to CP.

**Table 2.** MS2 virus-like particles as capsids for armored RNA.

Target	Expression system	Sequence length	Ref.
HIV	One-plasmid, 1 wt *pac* site	172 nt	[46]
Entero		Up to 500 nt	[50]
HCV			[51]
HCV			[60]
Entero			[61]
HCV			[62]
Lassa virus			[63]
SARS-CoV, H5N1, HCV	Two-plasmid 1× C5 variant *pac* site	2248 nt	[53]
SARS-CoV, HCV	One-plasmid 2× C5 variant *pac* site	1891 nt	[55]
HIV	One-plasmid, double expression 3× C5 variant *pac* site	3034	[56]
Measles virus		385	[57]
MERS		1002 (1629 + 394)	[58]
SARS-Cov-2		2908	[59]

HCV: Hepatitis C virus; MERS: Middle East Respiratory Syndrome; nt: Nucleotide.

### Therapeutic RNA

Therapeutic RNA can be classified into ncRNA, sRNA and coding mRNA. The former is further differentiated into siRNA, which is double stranded and inhibits expression of one specific mRNA, or miRNA (miR), which is single stranded and acts by binding to the 3′-untranslated region of a target mRNA, causing its degradation or translational suppression. By contrast, therapeutic mRNA is meant to be translated and expressed to produce a protein of interest. In all cases, the therapeutic RNA needs to be delivered to the living cells. MS2 VLPs, being natural protective shells for the phage RNA genome, seem to be the ideal tool for this purpose. Indeed, both sRNA and mRNA have been successfully encapsulated in MS2 VLP. While siRNA and miR can be relatively easily packaged, using the same strategy as for generation of the AR, efficient loading of functional mRNA remains more challenging.

The first report describing attempts to encapsulate miR in MS2 VLPs used the two-plasmid method and targeted a 385 nt sequence of the 5′-untranslated region of the HCV genome [64]. Even though such preparations were successfully delivered to Huh-7 cells, it is not clear how efficient the encapsulation itself was (the published work shows a limited effect on translation inhibition). In the subsequent years, MS2-encapsulated *miR-146* [65,66] and *miR-122* [67,68] have been reported, targeting, respectively, systemic lupus erythromatosis autoantibodies, osteoclast precursors and hepatocellular carcinoma. In these studies, a one-plasmid system with double *pac* site (C variant) was used for encapsulation. Importantly, the obtained constructs were not only successfully delivered to cells (*in vitro* and *in vivo*) by means of cell-penetrating peptides tethered to the external surface of MS2 VLP, but also demonstrated substantial translation inhibition. Similarly to AR, MS2 VLPs harboring miR can be affinity-purified using His-tag displayed on their surface, as shown by Mikel and coauthors, who adopted this idea for one-plasmid double-expression strategy [69].

A different approach was undertaken by Ashley and colleagues [70] who encapsulated siRNA (targeting *CCNA2* gene): they chemically disassembled MS2 VLPs by treating them with acetic acid and then reassembled them by restoring the neutral pH, in presence of siRNA of interest. The yield of this method was 85 molecules of siRNA per one MS2 VLP (as claimed by the authors). They also demonstrated the functional efficacy of cargo-loaded and cell-penetrating peptide-decorated particles delivered to cells in culture.

In the case of mRNA to be expressed in mammalian cells, two important sequence modifications are essential: capping the 5′-termini and polyadenylation of the 3′-termini. As neither of them can be conferred by bacteria, a eukaryotic expression system must be used for simultaneous mRNA production and encapsulation into MS2 VLP. For this, a one-plasmid double expression can be used in *Saccharomyces cerevisiae*. The feasibility of this strategy was first demonstrated by Legendre and Fastrez, who incorporated mRNA coding for human growth hormone (896 nt) into MS2 VLPs [71]. However, these authors confirmed the functionality of their construct only indirectly: by showing that mRNAs extracted from such MS2 preparations can be efficiently translated in cell culture. Eventually, another group managed to package mRNA coding for a prostate cancer antigen (1544 nt) into MS2 VLPs and showed that the resulting antigen is properly expressed by macrophages (following phagocytosis) [72]. Moreover, they demonstrated that the expressed antigen induces the expected immune response in a mice model, thereby providing a proof-of-concept for an MS2 VLP-based therapeutic vaccine.

Recently, an interesting example of chimeric MS2-alpharetrovirus Gag VLPs for encapsulation of the RNA components of the CRISPR-Cas system has been reported [73]. Namely, the authors of this work managed to package not only mRNA coding for the Cas enzyme but also sgRNA in chimeric particles. Furthermore, with such preparations, they were able to edit targeted genes in human primary cells. Impressively, they even showed that co-packaging of multiple sgRNAs into their chimeric VLPs allowed for the knockout of up to three genes.

Examples of therapeutic RNA packaged into MS2 VLPs are gathered in Table 3.

**Table 3.** MS2 virus-like particles as capsids for therapeutic RNA.

RNA type	Target	Expression system	Cell delivery strategy	Ref.
siRNA	Cyclin	Disassembly/reassembly (no *pac* site)	SP94 peptide HCC	[70]
miRNA	HCV IRES	Two-plasmid 1× *pac* site C-variant	TAT peptide Huh-7 cells	[64]
	miR-146a (SLE)	One-plasmid 2× *pac* site C-variant	TAT peptide HeLa, the human HCC cell line HepG2 and Huh-7 cells	[65] [74] [66]
	miR-122 (SLE, osteoporosis)	One-plasmid 2× *pac* site C-variant	TAT peptide HCC Hep3B, HepG2 and Huh7 cells and Hep3B	[67]
mRNA	Human growth hormone (896 bp)	One-plasmid (yeast) 1× wt *pac* site	None	[71]
	HIV-1 Gag mRNAs (1544 bp)	One-plasmid (yeast) 2× *pac* site C variant	Intramuscular vaccination (mice)	[72]
	Prostate acid phosphatase (1062 bp)	One-plasmid, 1× *pac* site C variant	Phagocytosis (murine macrophages) Intravenous vaccination (mice)	[75]
sgRNA (CRISPR)	SpCas9 mRNA and sgRNA targeting *TP53*/*Trp53* genes	Adopted to GagMS2 particles	Fibroblasts, hepatocytes, and cord-blood-derived stem and progenitor cells	[73]

HCC: Hepatocellular carcinoma; IRES: Internal ribosome entry site; SLE: Systemic lupus erythematosus.

**Figure 3.**
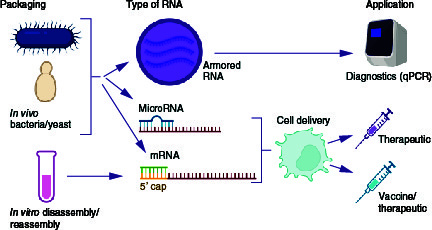
Reported developments in the field of RNA packaging into MS2 virus-like particles. Figure created with Biorender.com. VLP: Virus-like particle.

In summary, packaging strategies of RNA into MS2 VLPs can be differentiated into ‘*in vitro*’ (when already formed MS2 VLPs are disassembled and then reassembled in the presence of RNA of interest) and ‘*in vivo*’ (when RNA packaging occurs in the producing cell [bacterial or yeast] simultaneously with VLP assembly). Packaging of foreign RNA is feasible using several expression settings, as depicted in Figure 3. The encapsulated RNA can be further classified depending on its type (sncRNA or mRNA) and resulting application: as therapeutics (and potentially vaccine) or controls in diagnostics (Figure 3).

## Conclusion

During the last three decades, MS2 VLPs have emerged as a versatile tool for multiple applications. Due to their relative flexibility in terms of both capsid and internal cargo modifications, these particles can potentially act as universal vaccines combining mRNA packaging, antigen display and decoration with targeting molecules. This ease of functionalization, together with the good safety profile and cost–effectiveness, also makes them attractive candidates for the burgeoning field of RNA therapeutics. It is worth noting that the two areas discussed in this review (scaffolds for foreign peptides and protective shells for heterologous RNA) are not exhaustive: MS2 VLPs are also used as vehicles for chemical agents [70,76], proteins and gold nanoparticles [77], and oligodeoxynucleotides [78]. Another important field of MS2 application, which takes advantage of its controllable affinity to RNA, is in-cell visualization of endogenous RNA [79]. With further development, MS2 VLPs have the potential to play a crucial role in the advancement of several biotechnological and clinical applications.

## Future perspective

MS2 is well-characterized and has already found practical application as a diagnostic in the form of AR. The most exciting areas for future development are in vaccines and RNA-delivery systems.

For future vaccine development, an obstacle is that approaches to date have involved the insertion of relatively short antigenic peptides into an external loop. A superior immune response may be generated if epitopes consisting of larger, folded protein domains could be displayed. This may be addressed by post-assembly attachment of such complex antigens (using adapter systems such as SpyTag/SpyCatcher, sortase, biotin, etc.,) – examples of which are mentioned in the text. Another important consideration for use of recombinant VLPs as vaccines is the risk of contamination with bacterial toxins (lipopolysaccharide and others). This could be potentially avoided by adopting bacteria-free production systems. However, it is also worth mentioning that VLPs from phages are in general considered as safer than adenovirus-derived vehicles for instance, as in Lam and Steinmetz [80].

For progress in RNA delivery, particularly mRNA, the challenge is to find a method to efficiently disassemble and reassemble the CPs to achieve a high yield of cargo loading. This also will require significant reengineering of the protein. One step toward this goal has been achieved with the demonstration of MS2 VLPs engineered to form larger particles with greater capacity [6]. Another approach is production in a eukaryotic host in order to bypass problems associated with assembly/disassembly. This could allow in-cell encapsulation of mRNA containing all necessary elements and most likely will be more efficient in terms of packaging.

To effectively reengineer the CP to endow it with required new properties, computational and artificial intelligence-based approaches will be invaluable. These could include, for example, Rosetta Protein Design [81], ProteinMPNN [82], AlphaFold [83] and RFdiffusion [84]. These tools, some of them very recent developments, will provide exciting new possibilities for developing a new generation of MS2-based VLPs.
